# Laparoscopic completion gastrectomy in elderly patients with remnant gastric cancer: a case series

**DOI:** 10.1186/s40792-019-0610-0

**Published:** 2019-04-17

**Authors:** Masaki Kaihara, Satoru Matsuda, Eisuke Booka, Fumitaka Saida, Jumpei Takashima, Hanako Kasai, Koki Mihara, Atsushi Nagashima, Tomohisa Egawa

**Affiliations:** 1 Department of Surgery, Saiseikai Yokohamashi Tobu Hospital, 3-6-1, Shimosueyoshi, Tsurumi-ku, Yokohama-shi, Kanagawa 230-8765 Japan; 2 Department of Surgery, Keio University School of Medicine, 35 Shinanomachi, Shinjuku-ku, Tokyo, 160-8582 Japan; 3 Department of Surgery, Saiseikai Kanagawaken Hospital, 6-6, Tomiya-chou, Kanagawa-ku, Yokohama-shi, Kanagawa 221-0821 Japan

**Keywords:** Remnant gastric cancer, Laparoscopic completion gastrectomy, Elderly patients

## Abstract

**Background:**

Open completion gastrectomy (OCG) has been selected to treat remnant gastric cancer (RGC) due to severe adhesions and difficulty recognizing anatomical orientation after primary gastrectomy. In general, elderly individuals’ physiological reserves gradually decrease. Moreover, elderly patients (EPs) often have multiple complicating factors (i.e., frailty and comorbidities), leading to more postoperative complications after abdominal surgery. Recently, several trials revealed the advantages of laparoscopic surgery for EPs with gastric cancer in early recovery. However, there are limited studies investigating the use of laparoscopic completion gastrectomy (LCG) for RGC in EPs. This study aims to assess the efficacy of LCG in EPs aged ≥ 70 years. We compared the short- and long-term outcomes of LCG with those of OCG.

**Case presentation:**

Twenty-one EPs who underwent completion gastrectomy for RGC between 2007 and 2017 were enrolled and classified into two groups according to the surgical approach, namely the LCG (*n* = 6) and OCG (*n* = 15) groups. We adopted the G8 geriatric screening tool to comprehensively evaluate the EPs’ physical, mental, and social functions. Patient characteristics, clinicopathological characteristics, surgical outcomes, and survival were retrospectively reviewed and compared between groups.

**Results:**

There was no significant difference in the preoperative modified G8, indicating that the EPs’ backgrounds between the groups were comparable. Of note, blood loss during surgery was significantly reduced in the LCG group [median (range); LCG, 50 ml (20.0–65.0); OCG, 465 ml (264.5–714.0); *p* = 0.002]. The median number of retrieved lymph nodes in the LCG and OCG groups were 7 (range 4–10) versus 3 (range 1–6), respectively. There were no statistically significant differences in postoperative hospitalization, intake of solid food, and Clavien–Dindo grade ≥ II postoperative complications. In patients with a history of gastrectomy for gastric cancer in the LCG group, operative time tended to be longer in patients who underwent D2 lymph node dissection as primary surgery.

**Conclusions:**

LCG was comparable to OCG for the treatment of RGC in EPs with significantly reduced blood loss. While LCG should be selected with caution in patients who have undergone D2 lymph node dissection as primary surgery, it could be considered as a surgical procedure in EPs with RGC.

## Background

In the Japan Clinical Oncology Group (JCOG) 0912 trial, laparoscopic surgery (LS) for treating early-stage gastric cancer was shown to be a feasible procedure in terms of adverse events and short-term clinical outcomes [1]. Currently, LS has been established as a standard treatment [2]. In addition, the LS safety profile in distal gastrectomy with D2 lymphadenectomy for advanced gastric cancer is comparable to that reported for open surgery (OS) regarding postoperative morbidity and mortality rates [3].

For remnant gastric cancer (RGC), the surgical resection is indicated in numerous cases [4] and open completion gastrectomy (OCG) is ordinarily selected due to severe adhesions and difficulty recognizing the anatomical orientation after primary gastrectomy. On the other hand, as LS has been developed for gastric cancer, there is increasing evidence that the laparoscopic completion gastrectomy (LCG) can be a therapeutic option for RGC [5]. Moreover, we conducted a study at Saiseikai Yokohamashi Tobu Hospital showing the safety and the efficacy of LCG [6].

Elderly peoples require special consideration due to the decline in physical, physiological, and social functions [7]. Thus, a careful and safe surgical approach in elderly patients (EPs) is required. Two meta-analyses revealed LS advantages for treating EPs with gastric cancer in terms of early recovery and fewer complications [8, 9]. However, there are limited studies investigating the use of LS for the treatment of RGC in EPs.

In the present study, we reviewed RGC patients assigned to either the LCG or the OCG group over a 10-year period. Evaluating short- and long-term outcome, we aim to assess the efficacy of LCG in treating EPs.

## Case presentation

### Patients and methods

We retrospectively reviewed 21 patients aged ≥ 70 years who underwent completion gastrectomy for RGC between April 2007 and October 2017 at Saiseikai Yokohamashi Tobu Hospital. The patients were classified into two groups according to the surgical approach, namely the LCG (*n* = 6) and OCG (*n* = 15) groups. We adopted the G8 geriatric screening tool to comprehensively evaluate the EPs’ physical, mental, and social functions [10]. Since the score for one question pertaining to the self-perception of health was not obtained, the total score was calculated based on the remaining seven questions. In all patients, the clinical stage was evaluated according to the Japanese Classification of Gastric Carcinoma [11]. Postoperative complications were categorized using the Clavien–Dindo classification (C-D) [12]. In this study, complications classified as C-D grade ≥ II were considered postoperative complications. Patient characteristics, clinicopathological characteristics, surgical outcome, and survival were retrospectively reviewed and compared between the LCG and the OCG groups.

Both surgical approaches were presented to the patients, and either OCG or LCG was selected based on their preferences and their surgeons’ recommendations. All surgeries were performed by board-certified surgeons (Japan Surgical Society), and all LCG were performed by TE, who is a qualified surgeon according to the Endoscopic Surgical Skill Qualification System.

### Surgical procedures

**Fig. 1:**
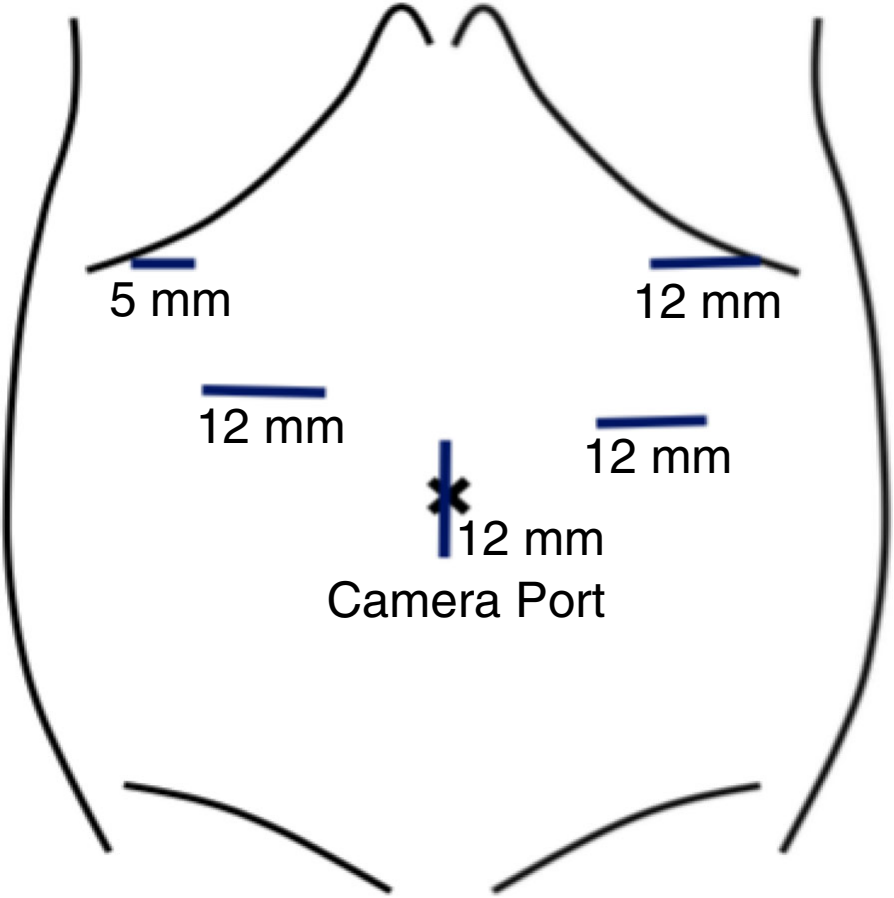
The placement of surgical ports

LCG and OCG with splenic preservation were conducted. Reconstruction was performed using the Roux-en-Y (R-Y) method. In LCG, patients under general anesthesia were placed in the reverse Trendelenburg position with the legs slightly apart. The surgeon stood on the patient’s left side, with an assistant on the right and another assistant holding a laparoscope between the legs. An initial 12-mm trocar was inserted at the umbilicus through the open method. Reviewing the abdominal cavity, we checked whether there were adhesions to the abdominal wall. In cases with previous LS, other ports were placed at the previous port wound (5 mm on the subcostal region and 12 mm on the abdomen bilaterally) (Fig. 1). A flexible electrolaparoscope was used, and the CO_2_ pressure was maintained at 10 mmHg.

The remnant stomach was completely mobilized by dissecting the adhesion between the remnant stomach and the left lateral segment of the liver, as well as the one between the remnant stomach and the pancreas. For patients in whom primary surgery was performed for benign disease, the supra pancreatic lymph nodes were resected in addition to those around the remnant stomach. The reconstruction was performed using the R-Y method through the pre-colon route. Esophagojejunostomy was performed using the overlap technique in the LCG group [13].

### Statistical analysis

Statistical analysis was performed using R version 3.5.1 (The R Foundation for Statistical Computing, Vienna, Austria). Demographic and surgical data were compared between the LCG and OCG groups. Continuous variables were analyzed using the Mann–Whitney *U* test. Categorical data were compared using Fisher’s exact probability test or the chi-squared test. Patient survival was calculated using the Kaplan–Meier method, and the significance of differences between the curves was analyzed using the log-rank test. A *p* < 0.05 denoted statistical significance in all analyses.

## Results

### Preoperative evaluation using the G8 geriatric screening tool

Using the G8 geriatric screening tool, we retrospectively evaluated the patients’ physical, mental, and social conditions. The answers to the individual questions are provided in Table 1. Two patients had lost > 3 kg of weight in the OCG group, whereas those in the LCG group tended to show a lower body mass index without statistical significance. Overall, there was no significant difference in the individual scores, resulting in total scores of 12.5 and 13.0 for the LCG and OCG groups, respectively (*p* = 0.841).

**Table 1:** Preoperative evaluation using the G-8 geriatric screening tool

Items (possible answers: score)	LCG*, n* = 6	OCG*, n* = 15	*p*
Decrease in food intake in the past 3 months			0.159
Severe: 0	0	0	
Moderate: 1	0	4 (27%)	
No decrease: 2	6 (100%)	11 (73%)	
Weight loss in the past 3 months			0.347
> 3 kg: 0	0	2 (13%)	
Does not know: 1	0	0	
Between 1 and 3 kg: 2	0	0	
No weight loss: 3	6 (100%)	13 (87%)	
Mobility			0.105
Bed or chair bound: 0	0	0	
Able to get out of bed and chair but does not go out: 1	1 (17%)	0	
Goes out: 2	5 (83%)	15 (100%)	
Neuropsychological problems			0.516
Severe dementia or depression: 0	0	0	
Mild dementia or depression: 1	0	1 (7%)	
No mental problems: 2	6 (100%)	14 (93%)	
Body mass index			0.182
< 19: 0	2 (33%)	1 (7%)	
19–21: 1	2 (33%)	5 (33%)	
21–23: 2	0	6 (40%)	
> 23: 3	2 (33%)	3 (20%)	
More than three prescribed medications			0.576
Yes: 0	2 (33%)	7 (47%)	
No: 1	4 (67%)	8 (53%)	
Self-perception of health	–	–	–
Not as good: 0			
Does not know: 0.5			
As good: 1			
Better: 2			
Age			0.125
> 85: 0	1 (17%)	0	
80–85: 1	2 (33%)	2 (13%)	
< 80: 2	3 (50%)	13 (87%)	
Modified score (median, range)	12.5, 11.3–13.8	13.0, 12.0–13.5	0.841

*LCG* laparoscopic completion gastrectomy, *OCG* open completion gastrectomy

### Preoperative patient characteristics

Comparisons of preoperative patient characteristics between the two groups are shown in Table 2. The proportion of males and patients who underwent OS as initial gastrectomy were significantly higher in the OCG group (*p* = 0.014 and *p* = 0.022, respectively). There were no differences between the two groups in terms of previous disease and reconstruction procedure. In addition, there was no significant difference in the time interval between the previous gastrectomy and the completion gastrectomy (*p* = 0.907).

**Table 2:** Preoperative patient characteristics

	LCG *n* = 6	OCG *n* = 15	*p*
Sex			0.014
Male	2 (33%)	13 (87%)	
Female	4 (67%)	2 (13%)	
ASA-PS class			0.526
I	4 (67%)	8 (53%)	
II	1 (17%)	1 (7%)	
III	1 (17%)	6 (40%)	
IV	0	0	
V	0	0	
ECOG-PS			0.105
0	1 (17%)	0	
1	5 (83%)	15 (100%)	
2	0	0	
3	0	0	
Original disease			0.291
Malignant	4 (67%)	13 (87%)	
Benign	2 (33%)	2 (13%)	
Previous approach			0.022
Open	3 (50%)	14 (93%)	
Laparoscopic	3 (50%)	1 (7%)	
Previous reconstruction			0.844
B-I	4 (67%)	8 (53%)	
B-II	1 (17%)	4 (27%)	
R-Y	1 (17%)	3 (20%)	
Time interval (years; median, range)	15.5, 2.3–37.8	7.0, 3.5–28.0	0.907

*LCG* laparoscopic completion gastrectomy, *OCG* open completion gastrectomy, *ASA-PS* American Society of Anesthetists-physical status, *ECOG-PS* Eastern Cooperative Oncology Group performance status, *B-I* Billroth-I reconstruction, *B-II* Billroth-II reconstruction, *R-Y* Roux-en-Y reconstruction

### Pathological findings, surgical outcomes, and postoperative course

In terms of surgical outcomes (Table 3), blood loss was significantly lower in the LCG group versus the OCG group [median (range); LCG, 50 ml (20.0–65.0); OCG, 465 ml (264.5–714.0); *p* = 0.002]. There was no significant difference in operative time between the two groups (*p* = 0.791). Due to the severe adhesion between the abdominal wall and small intestine, the laparoscopic approach was converted to the open approach in one patient from the LCG group who had undergone open gastrectomy with Billroth-I reconstruction for gastric cancer as primary surgery. The median number of retrieved lymph nodes in the LCG group was 7 (range 4–10) versus 3 (range 1–6) in the OCG group (*p* = 0.171). The LCG and OCG did not differ with respect to the distribution of TNM stages. Furthermore, there were no statistically significant differences in postoperative hospitalization (*p* = 0.410), intake of solid food (*p* = 0.867), and the occurrence of C-D grade ≥ II postoperative complications (*p* = 0.477). Thirty days after surgery, there was no reported perioperative mortality in either group.

**Table 3:** Pathological findings, surgical outcomes, and postoperative course

	LCG *n* = 6	OCG *n* = 15	*p*
Operative time (min; median, range)	310.5, 249.5–337.8	263.0, 241.5–325.0	0.791
Blood loss (ml; median, range)	50, 20.0–65.0	465, 264.5–714.0	0.002
Open conversion, *n* (%)	1 (17%)	0	0.105
Number of retrieved LNs (median, range)	7, 4–10	3, 1–6	0.171
pT			0.869
T1	2 (33%)	5 (33%)	
T2	0	0	
T3	1 (17%)	4 (26%)	
T4	3 (50%)	6 (40%)	
pN			0.642
N0	6 (100%)	13 (87%)	
N1	0	1 (7%)	
N2	0	1 (7%)	
N3	0	0	
pM			0.347
M0	6 (100%)	13 (87%)	
M1	0	2 (13%)	
pStage			0.562
IA	2 (33%)	5 (33%)	
IB	0	0	
IIA	1 17%)	3 (20%)	
IIB	0	0	
IIIA	3 (50%)	3 (20%)	
IIIB	0	0	
IIIC	0	2 (13%)	
IV	0	2 (13%)	
Postoperative hospitalization (days; median, range)	9, 7.3–13.8	9, 8.5–17.5	0.410
Intake of solid food (days; median, range)	4, 3.3–4.0	3, 3.0–4.0	0.867
Postoperative complication (C-D grade ≥ II)	3 (50%)	5 (33%)	0.477
Mortality within 30 days after surgery	0	0	–

*LCG* laparoscopic completion gastrectomy, *OCG* open completion gastrectomy, *LN* lymph node, *C-D* Clavien–Dindo

### Survival after completion gastrectomy

**Fig. 2:**
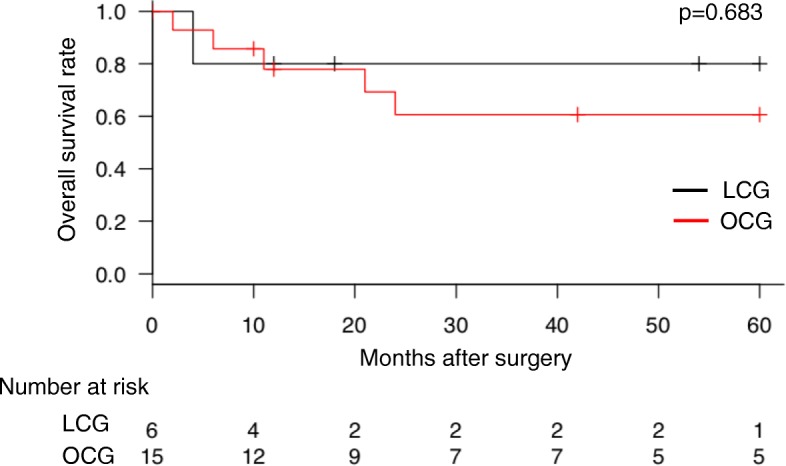
Overall survival. There was no significant difference in overall survival between the LCG and OCG groups. LCG laparoscopic completion gastrectomy, OCG open completion gastrectomy

In this study, the median follow-up period was 21 months, and the 5-year overall survival rate was 80.0% versus 60.6% in the LCG and OCG groups, respectively (*p* = 0.683, Fig. 2). Of the six deaths which occurred during the follow-up period, five were attributed to gastric cancer recurrence (one and four deaths, respectively). The remaining death was attributed to other diseases.

### Comparison of the modified G8 geriatric screening tool score in patients with or without postoperative complications

We evaluated the correlation between the modified G8 geriatric screening tool score and the occurrence of postoperative complications. The analysis showed that there was no significant difference in the modified G8 geriatric screening tool scores between patients with and without postoperative complications (*p* = 0.654, Table 4).

**Table 4:** Comparison of the modified G8 geriatric screening tool scores in patients with or without postoperative complications

	Postoperative complication (C-D grade ≥ II)		*p*
	Present (*n* = 8)	None (*n* = 13)	
Modified G8 geriatric screening tool score (median, range)	13, 11.0–14.0	13, 12.8–13.3	0.654

*C-D* Clavien–Dindo

### Comparison of the postoperative modified G8 geriatric screening tool score among the patients who did not exhibit postoperative recurrence

We also evaluated the role of G8 geriatric screening score 1 year after surgery. Based on the review of the clinical records, we could evaluate the scores of nine patients, including three and six patients in the LCG and OCG groups, respectively, among the 17 patients who did not exhibit postoperative recurrence. We found that there were no significant differences in any of the factors including the total score [median (range); LCG, 8.0 (7.5–8.5); OCG, 10.5 (8.3–12.8); *p* = 0.362].

### Surgical outcome in LCG patients with a history of gastrectomy for gastric cancer as primary surgery

In the LCG group, two of the four patients with a history of gastrectomy for gastric cancer underwent D2 lymph node dissection as primary surgery. As shown in Table 5, operative time tended to be longer in patients with D2 gastrectomy [median (range); D2, 352.0 min (349.0–355.0); D1 or D1+, 251.8 min (271.5–292.3); *p* = 0.333].

**Table 5:** Surgical outcomes in patients with the history of gastrectomy for gastric cancer as primary surgery in the LCG group

Lymphadenectomy for primary surgery	D2 (*n* = 2)	D1+/D1 (*n* = 2)	*p*
Operative time (min; median, range)	352.0, 349.0–355.0	271.5, 250.8–292.3	0.333
Blood loss (ml; median, range)	30, 20–40	190, 130–250	0.333
Open conversion, *n* (%)	0	1 (50%)	0.248
Postoperative hospitalization (days; median, range)	9, 8.5–9.5	12, 9.5–14.5	1
Intake of solid food (days; median, range)	4.0, 4.0–4.0	2.5, 2.4–2.8	0.221
Postoperative complication (C-D grade ≥ II)	1 (50%)	0	0.248
Mortality within 30 days after surgery	0	0	–

*C-D* Clavien–Dindo

## Discussion

There was no significant difference in the preoperative modified G8 geriatric screening tool score, indicating that the EPs’ backgrounds in each group were comparable. Blood loss during surgery was significantly reduced in the LCG group. In addition, there was no significant difference in oncological outcomes (e.g., the number of dissected lymph nodes) and prognosis. Overall, we demonstrated that LCG was shown to be applicable for treating EPs aged ≥ 70 years.

A standard surgical treatment for RGC has not been established. In terms of the indication of splenectomy, total gastrectomy without splenectomy was shown to be non-inferior to the one without splenectomy in treating proximal advanced gastric cancer without the lesions on the greater curvature in the JCOG 0110 trial [14]. However, because the lymphatic network is altered following primary surgery for RGC [15], the results of the JCOG 0110 trial are not applicable to treating RGC. Moreover, primary gastrectomy may influence completion gastrectomy, including severe adhesion development. Therefore, OCG has been preferable for treating RGC. The present study was consistent with this paradigm; OCG was frequently selected for patients who had undergone OS as primary surgery (Table 2).

Ohira recommended endoscopic surveillance after distal gastrectomy and detection RGC at an early stage. Moreover, the minimally invasive approach of endoscopic submucosal dissection and LCG may provide patients with an improved quality of life [4]. Currently, five studies comparing LCG and OCG in RGC patients have been reported [5, 16–19]. These studies have shown LCG is safe and effective for treating RGC, including reduced blood loss, increased number of dissected lymph nodes, shorter time to first flatus, and lower complication rate. Consistent with these results, we recently reported the advantages of LCG over OCG [6].

Elderly peoples’ physiological reserves gradually decrease. Moreover, elderly peoples’ cases are characterized by multiple complicating factors such as frailty and comorbidities [7]. Consequently, EPs experience more postoperative complications after abdominal surgery [20]. Therefore, minimally invasive and enhanced recovery approaches are warranted for treating EPs. In the current analysis, LCG was associated with reduced blood loss, versus OCG, without extending operative time. This finding demonstrated the favorable safety profile in LCG. Furthermore, there was no significant difference in oncological outcomes and prognosis between the LCG and OCG groups. On the other hand, in the LCG group, the operation time tended to be longer in patients who had D2 lymph node dissection for gastric cancer as primary surgery. Especially for elderly patients, the physiopathological implications of laparoscopy including prolonged operation time should be carefully considered [21]. Therefore, LCG needs to be cautiously selected in patients who underwent D2 lymph node dissection for gastric cancer as primary surgery.

The present study was limited to retrospective assessments. To minimize bias in patient recruitment, we reviewed consecutive EPs who underwent completion gastrectomy at Saiseikai Yokohamashi Tobu Hospital. Another limitation of this study is that the surgical approach was selected based on patient preference and surgeons’ recommendations. However, the retrospective evaluation, using the modified G8 geriatric screening tool score, confirmed no significant differences between the two groups (Table 1), indicating they were comparable in terms of vulnerability.

## Conclusions

LCG was shown to be comparable to OCG for treating RGC in patients aged ≥ 70 years. Furthermore, blood loss during surgery was significantly reduced in LCG versus OCG. While LCG should be selected with caution in patients who have undergone D2 lymph node dissection as primary surgery, it could be considered as a surgical procedure in EPs with RGC.

## Abbreviations

C-D: Clavien–Dindo
EPs: Elderly patients
JCOG: Japan Clinical Oncology Group
LCG: Laparoscopic completion gastrectomy
LS: Laparoscopic surgery
OCG: Open completion gastrectomy
OS: Open surgery
RGC: Remnant gastric cancer
R-Y: Roux-en-Y
